# Grade III Distal Medial Collateral Ligament Tear Missed by Magnetic Resonance Imaging: A Report of Two Cases

**DOI:** 10.7759/cureus.2251

**Published:** 2018-03-01

**Authors:** Vivek Tiwari, Dickey R Marak, Maximilian Muellner, Christoph Resinger, Thomas Muellner

**Affiliations:** 1 Department of Orthopaedics, All India Institute of Medical Sciences, Bhopal, India; 2 Orthopedics, Narayana Superspeciality Hospital; 3 Orthopaedics, Evangelisches Krankenhaus

**Keywords:** medial collateral ligament, sports injury, knee, ligament tear, magnetic resonance imaging

## Abstract

Medial collateral ligament (MCL) injuries are the most common knee ligament injuries. Magnetic resonance imaging (MRI) is the investigation of choice for detecting such injuries. We report two cases of acute grade 3 MCL tears in young adults in which the injury was suspected clinically and was later confirmed by surgical exploration. However, the MRI failed to pick up the exact nature of injury. This report signifies the importance of an appropriate clinical examination for MCL injuries and stresses that decision-making for treatment should be based on the clinical examination rather than the MRI.

## Introduction

Sports injuries are common orthopedic events in the young population. Knee injuries are considered the most frequently encountered sports-related injuries. During such injuries, some of the structures at risk include the anterior cruciate ligament (ACL), posterior cruciate ligament (PCL), menisci, lateral collateral ligament (LCL), and medial collateral ligament (MCL). Magnetic resonance imaging (MRI) is the investigation of choice to confirm injury to knee ligaments, including the MCL [1]. Although an MRI is not 100% sensitive for detecting MCL injuries [2], surgeons can rely on its findings for a diagnosis in grade 3 MCL tears. We report two cases of acute grade 3 MCL tears in young adults in which the injury was suspected clinically and later on confirmed by surgical exploration. However, the MRI failed to pick up the exact nature of injury.

## Case presentation

Patient 1

**Figure 1:**
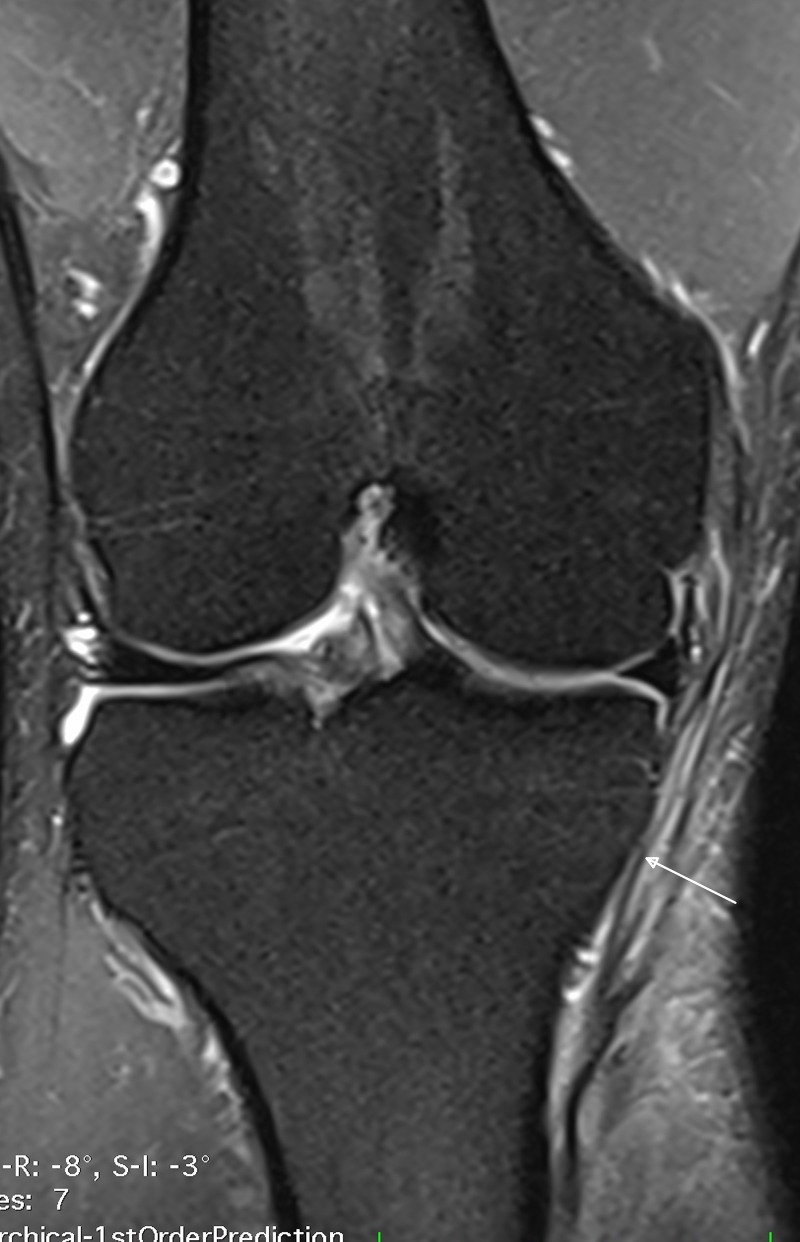
Magnetic resonance imaging of the right knee for patient 1 The T2 weighted image shows the continuity of the fibers of the superficial band of the MCL (white arrow).

A 22-year-old man presented to the outpatient department with complaints of pain in the right knee following a history of a twisting injury during a soccer game five days ago. It was associated with minimal swelling and instability in the right knee. On examination, there was tenderness at the medial aspect of the right proximal leg corresponding to the tibial insertion site of the MCL. The valgus stress test was negative on 0º and grade 3 positive in 30º knee flexion. The tests for anterior instability, posterior instability, varus stress test, and McMurray’s test for both menisci were negative. There was no distal neurovascular deficit. He was diagnosed to have a grade 3 tear of the right MCL. Plain radiographs of the right knee (anteroposterior and lateral views) were unremarkable. An MRI of the right knee revealed minimal joint effusion with intact ACL, PCL, LCL, MCL, and both menisci (Figure 1).

Based on his symptoms and positive valgus laxity, he was planned for MCL exploration and repair. Under general anesthesia, a five-cm skin incision was made over the medial aspect of the right proximal leg. Subcutaneous tissue was incised in the line of the skin incision and superficial MCL was explored after opening the sartorius fascia. The superficial MCL was found completely torn from its tibial attachment. The meniscotibial and meniscofemoral parts of the MCL (deep MCL) were intact. The superficial MCL was subsequently repaired with internal bracing using Fibertape (Arthrex, Naples, FL) and suture anchors (SwiveLock 4.5-mm-diameter PEEK anchors; Arthrex, Naples, FL). Post-operatively, the knee was immobilized in an above-knee brace for three weeks followed by a gradual range of motion exercises. At a follow-up of six months, the knee was completely stable and pain-free with full range of motion.

Patient 2

**Figure 2:**
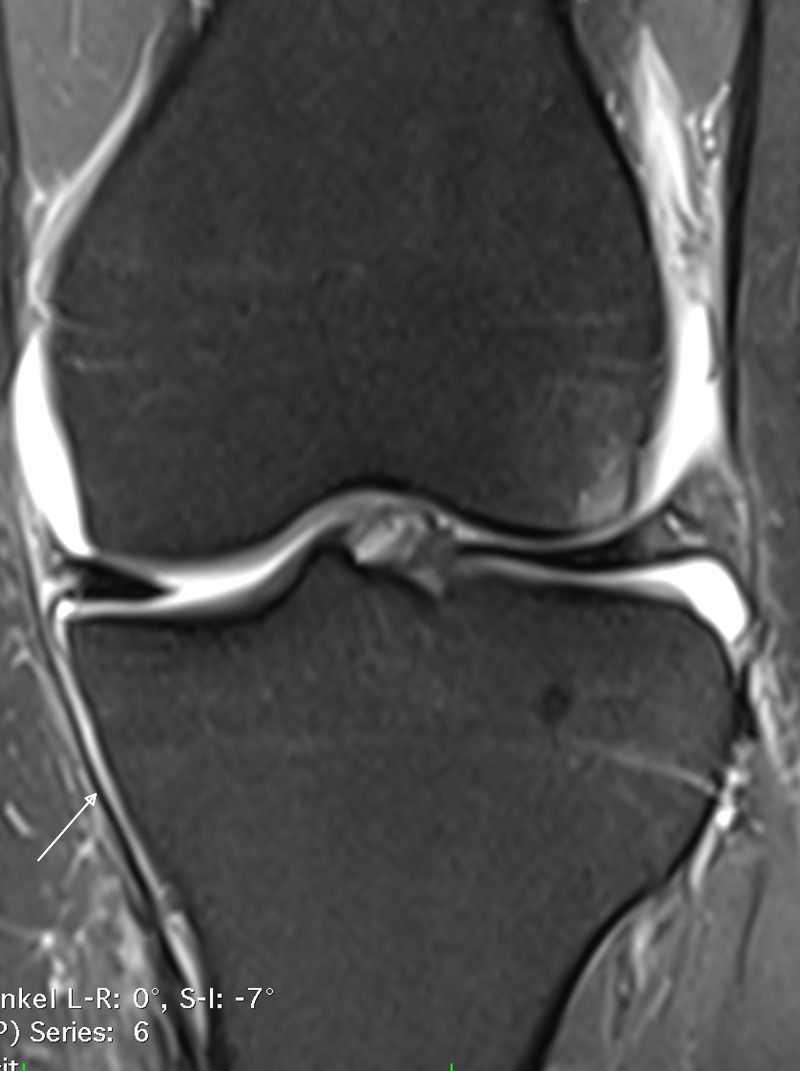
Magnetic resonance imaging of the left knee for patient 2 The T2 weighted image shows the intact superficial band of the MCL (white arrow).

Another 22-year-old female professional basketball player presented to us with complaints of pain in the left knee associated with anterior instability for one week following a twisting injury to the left knee. There was associated mild knee swelling. On examination, the patient had tenderness at the medial aspect of the left proximal leg corresponding to the tibial insertion site of the MCL. The anterior drawer test and Lachman’s test for ACL were positive. The valgus stress test was grade 3 positive on 0º as well as 30º knee flexion. There were no associated clinical signs of varus laxity, posterior instability, and medial or lateral meniscus tear and the distal neurovascular status was normal. Plain radiographs of the left knee (anteroposterior and lateral views) were normal. She was diagnosed to have a complete ACL tear with a grade 3 tear of the left MCL. MRI left knee revealed mild joint effusion and complete ACL tear with the other structures, including MCL intact (Figure 2).

**Figure 3:**
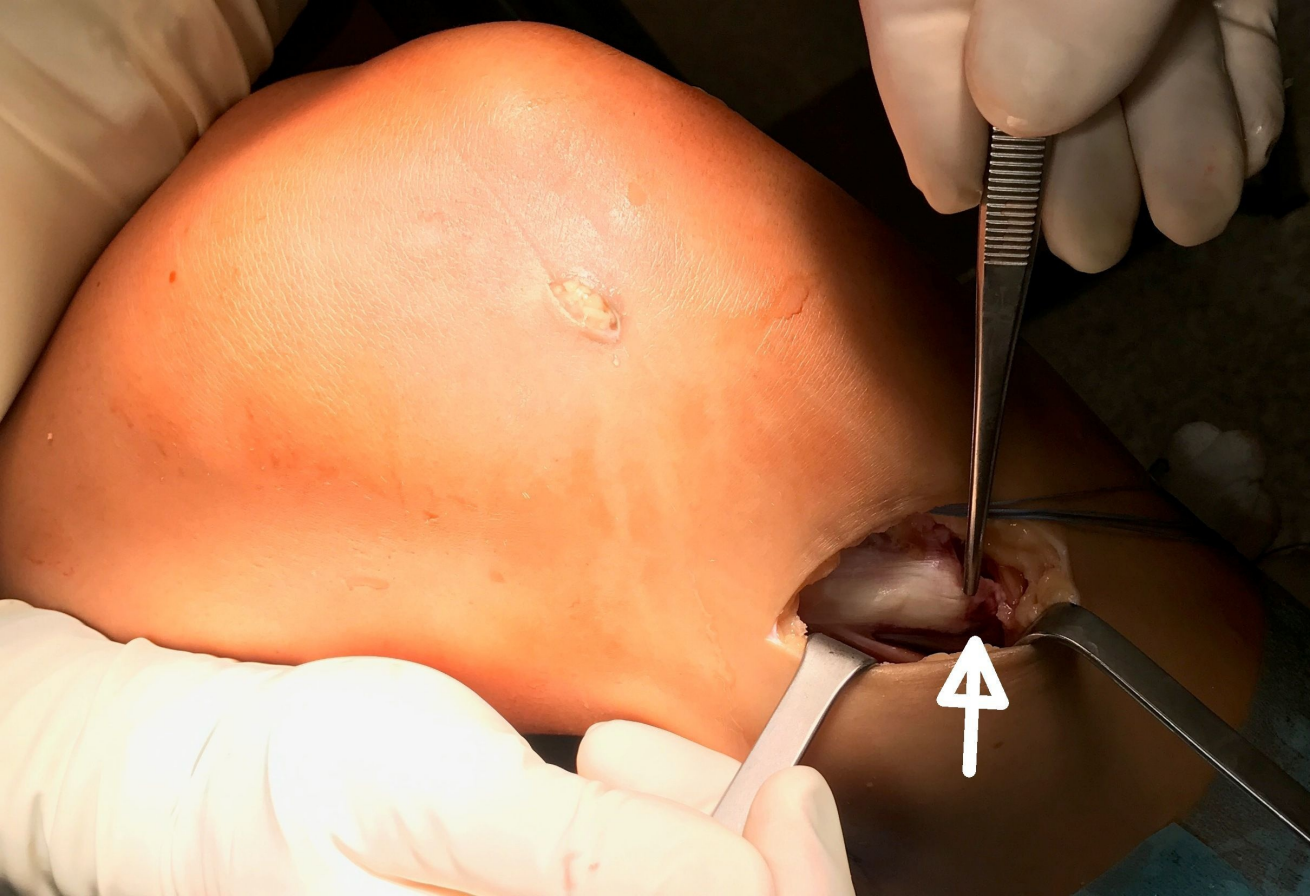
An intraoperative photograph of patient 2 The superficial band of the MCL is seen detached from its tibial attachment (white arrow).

She was planned for ACL reconstruction and repair of the MCL based on the symptoms and clinical signs. After arthroscopic ACL reconstruction using semitendinosus-gracilis (STG) tendons, the incision used for graft harvest was used to explore the MCL. The superficial MCL was found completely torn from its tibial attachment (Figure 3).

The meniscotibial and meniscofemoral parts of the MCL (deep MCL) were found intact. Subsequently, the superficial MCL was repaired with a Suture Tape (Arthrex, Naples, FL) and suture anchors (PushLock 3.5-mm-diameter PEEK anchors; Arthrex, Naples, FL) at each corner of the torn MCL. Post-operatively, the knee was immobilized in an above-knee brace for six weeks with a gradual range of motion exercises. At a follow-up of six months, the knee was completely stable and pain-free with full range of motion.

## Discussion

MCL injuries have been reported as the most common knee ligament injury [3]. The MCL consists of a two-layered anterior part and a posterior part. The anterior part has a superficial band and a deep band (meniscofemoral and meniscotibial fibers) [1]. Injury to the knee ligaments, including the MCL, can be best seen on MRI. There can be various presentations of MCL injuries on MRI. However, certain injury findings in the knee can be potentially missed on MRI [4]. Also, low-grade injuries of MCL can be overestimated on MRI due to a similar presentation in other conditions, such as a medial meniscal tear, medial cellulitis, medial meniscal cyst, MCL bursitis, and medial osteoarthritis [1,4-5]. Similarly, low-grade injury to the deep band of MCL can sometimes be underreported [4]. However, it is very rare for a tear of the superficial band of MCL to be missed on MRI. Furthermore, there is no report in the literature about any grade 3 tear of MCL not to be picked up on MRI. Although Halinen et al. have reported a 86% sensitivity and accuracy of MRIs for the type of MCL injury in a series of 21 patients with a multi-ligament injury of the knee, the MRI was able to detect at least grade 2 tears in all the cases [2].

We report two cases of a complete tear of the MCL from its tibial attachment (grade 3 tear), which was not evident on MRI. Based on the clinical findings and symptoms, we decided to explore the MCL, confirming the MCL injury, which was subsequently repaired. Therefore, MRI findings should not always be relied upon in case of MCL injuries.

## Conclusions

Our findings signify the importance of a thorough clinical examination for knee ligament injuries. The surgeon should make a clinical diagnosis based on his examination findings and the MRI should only be used to confirm it. In case of a discrepancy between the two, one should always rely on the clinical findings to decide treatment plans.
